# Merc. Corr. in Dysentery, Etc.

**Published:** 1883-02

**Authors:** Carroll Dunham


### MERC. CORR. IN DYSENTERY WITH URINARY COMPLICATIONS.

CARROLL DUNHAM, M. D.

This interesting case of Dr. Dunham's, reported by Dr. Wells, shows us in what cases Merc. corr. *is the* remedy for dysentery. Dr. Dunham's testimony also goes to prove that Merc. corr. is seldom called for in this disease, and is in no sense a specific for it. Dr. Dunham writes: "During a practice of several years, in which dysentery was not unfrequently met, and often in severe forms, I never prescribed Merc. corr. in a single case, nor do I recollect a single case in which I think it could have been given with advantage or with the result of expediting the cure at all.

"With Merc. sol., Nux v., Coloc., Caps., Sulph., and Lachesis, I have always succeeded, as it appeared to me, in curing as rapidly as was possible. And observations of cases under the treatment of my colleagues, some of whom gave Merc. corr. and Coloc. as a standard prescription for dysentery, satisfied me that the rapidity and ratio of my cures were, at the very least, as satisfactory as theirs.

"During the summer of 1861, however, two cases came under my treatment which presented certain features heretofore unobserved by me, and in which none of the remedies above named was clearly indicated and none did any good, but which both yielded most promptly to Merc. corr.

"The first was a widow of twenty-five years, who had just lost her only child of typhoid fever. When I was called to her she lay in bed, with constant heat of skin; quick, rather small, pulse; tongue dry, with a yellow coat; thirst not excessive; abdomen sore on pressure, somewhat distended; constant, uninterrupted pressure to stool felt in the sacral and the hypogastric regions. This pressure, though very distressing to the patient, seemed utterly ineffectual, so far as the evacuations of the bowels were concerned. It was not worse before a stool and no better after a stool; the stool consisted of a bloody slime in small masses. There was great tenesmus vesicæ, the urine was scanty, hot, and bloody; the disposition was quiet, slightly desponding—little disposition to sleep; on the whole, the suffering, which was evidently severe, was endured with much patience, and this was a decided contra-indication for Arsenic, between which and Merc. sol. my choice was at first divided. The general symptoms certainly did not correspond with the characteristics of Arsenicum. The character of the thirst, the absence of great restlessness, of nocturnal aggravation of a paroxysmal character, of prostration greatly out of proportion to the severity of the symptoms—all contra-indicated Arsenicum. Mercurius sol. has pains of a paroxysmal character. It lacks the constant tenesmus and tenesmus vesicæ. I gave it, however, and in various potencies, but it exercised no influence on the disease. Belladonna, Colocynthis, and finally Arsenic, were of no avail. Indeed, I gave them without confidence of a favorable result, for it was evident they were not indicated.

"Remembering, now, Hahnemann's remarks in the introduction to the proving of Merc. sol.,* 'I have found a very small drop of the quintillionth, or, still better, of the decillionth potency of Merc. corr., given *alone in a single dose,* almost a specific in the ordinary autumnal dysentery,' I turned to the few symptoms of Merc. corr., which Hahnemann gives in the same volume of the *Materia Medica Pura,* and found among others, which corresponded to the case in hand, the following symptoms:

"'24. With almost constant cutting pains in the abdomen, and intolerable, painful, almost ineffectual pressing, straining, and tenesmus, frequent scanty discharge of bloody slime, day and night.'

"'17. Immediately after stool, pressing downward in the front of the abdomen, below the umbilicus.'

"'28. Tenesmus vesicæ.'

"I now concluded to give Merc. corr., and remembering Hahnemann's injunction that those who follow his directions in the hope of gaining results like his should follow them *accurately* ('Machen sie nach, machen sie eber, richtig, nach'), I gave one single dose of Merc. corr. at eight P. M., and awaited the result. This was in every way so satisfactory that I gave the patient no more medicine at all. She was clearly convalescent on the following day, having no more of the characteristic pains and stools of dysentery, no more fever, and being able to begin to take nourishing food. After two days of pure expectant observation I was able to dismiss her cured.

"The second case, which presented itself to me within the same month, was similar in all respects, though much less severe as regards intensity. I gave Merc. corr.$^{30}$, as before, in a single dose, and the patient was convalescent the next day.

---

* *Materia Medica Pura,* Vol. I, p. 355, German edition.

"Since this period I have treated about twenty-eight cases of well-marked dysentery, but have had no difficulty in curing them with Nux vom., Merc. sol., or Coloc., and have seen no similarity in them to those in which Merc. corr. had been so successful.

"Since the above was written, an exception to the stated want of success with remedy in the treatment of dysentery has occurred in the practice of the writer, in *which suppression of urine and tenesmus of the bladder* were prominent symptoms. The case was promptly cured.".

NOTE: We desire to call attention to two points in the above quotation from Dr. Dunham. He says: "During a practice of several years, in which dysentery was not unfrequently met, and often in severe forms, *I never prescribed Merc. corr.* in a single case"!! (Italics ours.) Again, Dr. Dunham quotes Hahnemann as saying: "I have found a very small dose of the quintillionth, or, still better, of the decillionth potency of Merc. corr., given *alone in a single dose*, ALMOST* a specific in the ordinary AUTUMNAL dysentery." Here Hahnemann places two conditions to his assertion that Merc. corr. is a specific for dysentery. He says it is "almost" a specific for "autumnal" dysentery. These limitations Dr. Hughes and others apparently have not noticed, for we read in the *British Journal of Homœopathy*, January, 1882, p. 75: "Dysentery * * Hahnemann tells us can always be cured by one remedy, viz., *Mercurius corrosivus*." *As we have seen, Hahnemann said no such thing;* nor does experience prove the remedy to be a specific.

Will the *British Journal* kindly elucidate?

* We put these words [illegible] that the apparently myopic Dr. Hughes may read it!

